# The role of warm, dry summers and variation in snowpack on phytoplankton dynamics in mountain lakes

**DOI:** 10.1002/ecy.3132

**Published:** 2020-09-16

**Authors:** Isabella A. Oleksy, Whitney S. Beck, Roderick W. Lammers, Cara E. Steger, Codie Wilson, Kyle Christianson, Kim Vincent, Gunnar Johnson, Pieter T. J. Johnson, J. S. Baron

**Affiliations:** ^1^ Natural Resource Ecology Laboratory Colorado State University Fort Collins Colorado 80526 USA; ^2^ Cary Institute of Ecosystem Studies Millbrook New York 12545 USA; ^3^ Department of Biology Colorado State University Fort Collins Colorado 80526 USA; ^4^ College of Engineering University of Georgia Athens Georgia 30602 USA; ^5^ Department of Geosciences Colorado State University Fort Collins Colorado 80526 USA; ^6^ Department of Fish, Wildlife, and Conservation Biology Colorado State University Fort Collins Colorado 80526 USA; ^7^ Department of Ecology and Evolutionary Biology University of Colorado Boulder Colorado 80309 USA; ^8^ Department of Geology Portland State University Portland Oregon 97201 USA; ^9^ U.S. Geological Survey Fort Collins Colorado 80526 USA

**Keywords:** alpine, climate change, cryosphere, limnology, mountain lakes, nitrogen deposition, phytoplankton, snowmelt timing

## Abstract

Climate change is altering biogeochemical, metabolic, and ecological functions in lakes across the globe. Historically, mountain lakes in temperate regions have been unproductive because of brief ice‐free seasons, a snowmelt‐driven hydrograph, cold temperatures, and steep topography with low vegetation and soil cover. We tested the relative importance of winter and summer weather, watershed characteristics, and water chemistry as drivers of phytoplankton dynamics. Using boosted regression tree models for 28 mountain lakes in Colorado, we examined regional, intraseasonal, and interannual drivers of variability in chlorophyll *a* as a proxy for lake phytoplankton. Phytoplankton biomass was inversely related to the maximum snow water equivalent (SWE) of the previous winter, as others have found. However, even in years with average SWE, summer precipitation extremes and warming enhanced phytoplankton biomass. Peak seasonal phytoplankton biomass coincided with the warmest water temperatures and lowest nitrogen‐to‐phosphorus ratios. Although links between snowpack, lake temperature, nutrients, and organic‐matter dynamics are increasingly recognized as critical drivers of change in high‐elevation lakes, our results highlight the additional influence of summer conditions on lake productivity in response to ongoing changes in climate. Continued changes in the timing, type, and magnitude of precipitation in combination with other global‐change drivers (e.g., nutrient deposition) will affect production in mountain lakes, potentially shifting these historically oligotrophic lakes toward new ecosystem states. Ultimately, a deeper understanding of these drivers and pattern at multiple scales will allow us to anticipate ecological consequences of global change better.

## Introduction

Globally, lakes are warming as a result of increasing air temperatures and reduced cloud cover (O’Reilly et al. 2015). Changing lake thermal regimes are subsequently driving additional changes in biogeochemical, metabolic, and ecological functions (Gerten and Adrian 2002, Kraemer et al. 2016). The indirect effects of warming, such as those caused by earlier ice‐out dates, can further alter lake dynamics by lengthening the growing season, which can alter phytoplankton populations and successional patterns (Schindler et al. 1990, George et al. 2004). However, the responses of specific water bodies to similar climatic drivers are likely to vary even within a single region, owing to differences in adjacent land cover, lake morphometry, and connectivity to other water bodies (Kraemer et al. 2015).

Mountain lakes are particularly vulnerable to warming trends (Pepin et al. 2015, Schmeller et al. 2018), but our knowledge of how primary producers will respond is limited. Until recently, changes in mountain lake phytoplankton have been attributed to nitrogen and phosphorus deposition, particularly in western North America (Goldman 1988, Wolfe et al. 2003, Brahney et al. 2015), but increases in mountain lake productivity are beginning to be described in the literature as a consequence of multiple concurrent stressors (Oleksy et al. 2020). The length of the ice‐free season is increasing, affecting lake thermal structure, solute concentrations, mixing regimes, and ultimately phytoplankton biomass and productivity (Roberts et al. 2017, Peter and Sommaruga 2017). In Arctic lakes, climate change is implicated as the primary driver of altered primary producer assemblages and ecosystem production through changes in ice cover and lake thermal structure (Ruhland et al. 2008, Griffiths et al. 2017). Similar processes are likely at work in mountain lakes but may be obscured by inputs of nutrients; where both warming and enrichment occur, such interactions may enhance current and future algal abundance in lakes (Jeppesen et al. 2014, Lepori et al. 2018).

Much of our understanding about variation in lake processes has emerged from a legacy of research in the northern and midwestern United States and northern European lake districts, which have distinctly different climate and land‐use characteristics compared to mountain lakes. Although this research has provided insight into the drivers of nutrient concentrations (Soranno et al. 2015), gross primary production (Kelly et al. 2018), phytoplankton–nutrient relationships (Wagner et al. 2011), and synchrony in responses across these landscapes (Magnuson et al. 2004), a thorough understanding of the patterns and drivers of phytoplankton dynamics in mountain lakes is lacking. Understanding the dominant drivers that regulate the base of lake food webs is critical for developing climate adaptation and biological conservation strategies, especially because these systems give rise to the major rivers of the world and support downstream communities (Huss et al. 2017, Klein et al. 2019). Mountain lakes can serve as model systems for understanding spatiotemporal ecosystem dynamics and processes affecting lake systems globally because of their responsiveness to environmental change and relatively undisturbed catchments (Moser et al. 2019).

There are an estimated 2,600 natural lakes 2,700 or more meters above sea level in the southern Rocky Mountains (SRM) (Nelson 1988). In light of recent increases in lake productivity observed in two SRM lakes stimulated by increased nutrients and warming (Oleksy et al. 2020), the goal of the current paper was to construct predictive models to describe phytoplankton biomass (as chlorophyll *a*) dynamics across multiple spatial and temporal scales in the region. Specifically, we used three data sets to ask (1) what are the most important drivers of phytoplankton biomass across the region? and (2) do the drivers of phytoplankton biomass differ interannually and intraseasonally? For the first question, we hypothesized that variation in phytoplankton biomass from lake to lake would be controlled by nutrient concentrations as well as watershed features that influence nutrient delivery in headwater aquatic ecosystems, including glacier, vegetation cover, and underlying geology (Ren et al. 2019). Land cover influences water quality and ecosystem functioning in other regional‐scale studies because of nutrient delivery from the adjacent landscape (Wagner et al. 2011, Filstrup et al. 2014, Lapierre et al. 2017). For the second question, we hypothesized that variation in annual snow‐water equivalent (SWE) would explain phytoplankton responses over decadal time scales, given the importance of SWE on growing‐season length in mountain lakes (Preston et al. 2016). Within a season, we expected that the role of nutrients, particularly the relative availability of nitrogen (N) to phosphorus (P), would explain the most variability in phytoplankton biomass, because the SRM region has been subjected to high atmospheric N deposition (Wolfe et al. 2003, Elser et al. 2009a).

## Methods

### Data acquisition

**Fig. 1:**
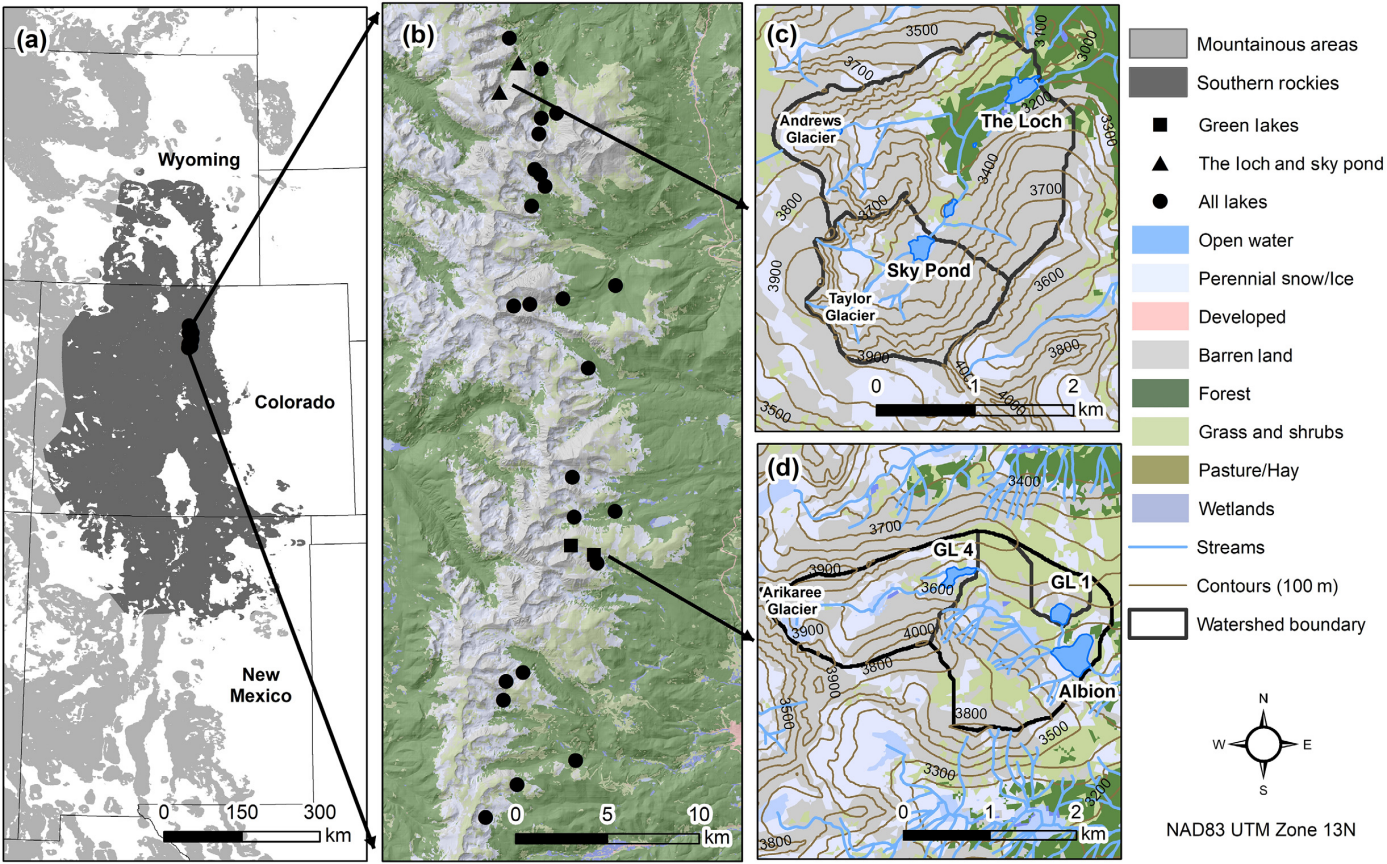
Locations of the study region and lakes included in the study: (a) the southern Rocky Mountain ecoregion (after the Western Lakes Survey; Eilers et al. 1987); (b) lakes included in the regional model; (c) the Loch Vale Watershed lakes (The Loch and Sky Pond; LVWS) that were included in the intraseasonal model; and (d) the Green Lakes Valley lakes (GL1 and GL4; GLV) that were included in the long‐term model.

To identify drivers of regional variation in phytoplankton biomass, 28 lakes from just below and above treeline (2,987–3,550 m) in the Colorado Front Range were sampled shortly after ice‐off and again during late summer between 2015 and 2016 (*n* = 147; Fig. 1). The majority were situated in watersheds with less than 25% vegetation cover (Table 1). The lakes were characteristic of SRM lakes according to the Western Lake Survey (Eilers et al. 1987), being on average ≤7 ha in surface area and ≤10 m deep in watersheds ≤400 ha or smaller (Appendix S2: Table S2). To identify drivers of interannual phytoplankton biomass, two alpine lakes, Green Lake 1 (GL1) and Green Lake 4 (GL4) from the Green Lakes Valley, part of the Niwot Ridge Long‐Term Ecological Research Program, were sampled a minimum of five times annually between ice‐off (May to June) and September between 2008 and 2016 (*n* = 104; Fig. 1). Finally, to identify drivers of intraseasonal phytoplankton biomass, the subalpine Loch and alpine Sky Pond within the Loch Vale watershed (LVWS) of Rocky Mountain National Park were sampled approximately weekly in 2015–2016 and monthly in 2017 from the week of ice‐off through mid‐September (*n* = 81, Baron 1992; Fig. 1).

**Table 1:** Summary information for predictor variables that were candidates in the best regional climate, regional climate + watershed, interannual, and intraseasonal models. Summer statistics include minimum, maximum, mean, median, and standard deviation for each predictor variable. Randomly selected monthly observations from Loch Vale watershed and Green Lakes Valley lakes are included in the model and data summary presented. Dashes indicate data were unavailable for all lakes or summary statistics could not be computed on categorical variables. DIN:TP data were only available for Loch Vale lakes. Twenty‐two land‐cover predictors were included in the original models but were dropped in the model selection procedure. Methods for land cover and summary of parameters can be found in Appendix S1: Table S1.

Variable	Description [units]	Minimum	Maximum	Mean	SD
Indexing variables					
DOY	Day of year	152	266	205	–
Year	Year	–	–	–	–
Climate variables					
Weekly precipitation	Cumulative precipitation for week preceding sample date [mm]	0.0	27.0	7.1	6.3
Monthly precipitation	Cumulative precipitation for 30 d preceding sample date [mm]	8.9	114.9	37.5	20.1
Precipitation % normal	Monthly precipitation as a percent of normal [%]	23%	122%	56%	33%
Daily mean temperature	Mean air temperature sample date [°C]	5.6	16.4	11.7	2.3
Monthly mean temperature	Mean air temperature for the 30 d preceding sample date [°C]	2.4	14.5	10.9	2.0
Temperature % normal	Monthly average air temperature as a percent of normal [%]	86%	171%	123%	21%
Maximum SWE	Maximum observed SWE for the preceding winter [in.]	4.5	21.8	14.9	7.1
Change snow (1992–2011)	Change in perennial snow and ice cover 1992 to 2011 [%]	−3.5%	0.0%	−1.1%	0.9%
Environmental variables					
NO_3_	Nitrate‐N [mg/L N]	0.002	0.40	0.09	0.07
DIN:TDP	Total dissolved N to total dissolved P molar ratio	18.1	1,287.4	167.4	153.5
DIN:TP	Total dissolved N to total P molar ratio	–	–	–	–
Water temperature	Water temperature of sample [°C]	2.6	19.0	9.4	3.3
Watershed variables					
Maximum lake depth	Maximum lake depth [m]	1.8	42.0	10.7	8.6
Drainage ratio	Lake area as a percentage of watershed area [%]	0.5%	10.2%	3.1%	2.7%

Predictor variables were collected as described in the following corresponding sections and classified as environmental, climatic, or watershed (Table 1, Appendix S1: Table S1). With each field visit, water samples were collected to analyze chlorophyll *a* concentrations (as a proxy for phytoplankton biomass) along with nutrient contents and temperature. We used three distinct data sets to produce our models:

1. Data from 28 lakes sampled from 2015 to 2016 (regional model);
2. Data from Green Lakes 1 and 4 from 2008 to 2016 (long‐term model);
3. Data from The Loch and Sky Pond from 2015 to 2017 (intraseasonal model).

Because of the high number of samples from The Loch, Sky Pond, and Green Lakes 1 and 4, we randomly selected one sampling date per month for each site to prevent these four lakes from disproportionately influencing the regional model results.

### Environmental variables

Water chemistry and chlorophyll *a* were collected at the deepest point of each lake from the upper mixed layer and hypolimnion with a peristaltic pump. Chlorophyll *a* samples were filtered (0.7 µm) in situ, held on ice until being returned to the laboratory, and then frozen until analysis. Water chemistry measurements included nitrate (NO_3_), total dissolved phosphorus (TDP), total dissolved nitrogen (TDN), and dissolved organic carbon (DOC). All samples were filtered within 24 h of collection and frozen until analysis. We only collected unfiltered aliquots for total phosphorus (TP) analysis for LVWS lakes. Water temperature and conductivity were measured in situ with a hand‐held probe (Thermo Scientific Orion 3‐Star, Waltham, Massachusetts, USA). Fish presence or absence data were based on investigator site‐specific knowledge or through fish stocking records from Colorado Parks and Wildlife. We included sampling depth as a predictor in the models to account for differences in drivers between epilimnion and hypolimnion samples. A full description of water‐chemistry lab methods is outlined in Appendix S1.

### Climate and weather variables

We used the *prism* package (Hart and Bell 2015) in R version 3.5.0 (R Development Core Team 2018) to obtain estimates of temperature and precipitation for each study site from the parameter‐elevation regressions on independent slopes model (PRISM Climate Group 2018). For each sample date, we extracted the daily mean temperature and total precipitation and calculated the mean daily temperature and total precipitation for the 7 d and the 30 d preceding the sampling date. To complement these data, we compared monthly temperature and precipitation to climate normal data (1981–2010) for the calendar month closest to the sampling date. We also obtained snowfall data for the winter preceding sampling from the nearest snow telemetry (SNOTEL, U.S. Department of Agriculture), including the maximum observed snow water equivalent, comparisons of this maximum SWE to average historical SWE (1980–2010 data), and the difference between the observed spring snow‐free date and historical average snow‐free date.

### Watershed variables

Watersheds for each lake were delineated from lake outlets with the U.S. Geological Survey (USGS) StreamStats online tool (USGS 2016). We calculated and extracted several watershed predictors (WS) that we hypothesized might play a role in explaining lake‐to‐lake variation in chlorophyll *a*, including dominant vegetation types, wetland extent, rock glacier and perennial ice cover, and underlying geology. A full description of how watershed variables were derived is included in Appendix S1: Table S1.

### Statistical analyses

We used boosted regression trees (BRTs) to identify drivers of chlorophyll *a* regionally, seasonally, and intra‐annually (Breiman et al. 1984, Elith et al. 2008). Regression trees provide flexibility by allowing for nonlinear relationships between predictor and response variables; are robust to missing predictor data, nonindependence, and collinearity; can detect interactions among predictors; and are often well‐suited for hierarchically structured predictor variables (De’ath and Fabricius 2000, Elith et al. 2008, Buston and Elith 2011). A key feature of BRT is recursive partitioning, which splits the response variable into groups that are as homogenous as possible based on predictor variable values (Strobl et al. 2009). BRTs combine recursive partitioning with boosting, a method for combining hundreds to thousands of trees to improve model performance and predictive capacity (Prasad et al. 2006, De’ath 2007, Elith et al. 2008).

We built three sets of BRT models with data collected from 28 southern Rocky Mountain lakes in Colorado, across a gradient of elevations, catchment types, land cover, and lake sizes (Fig. 1). The first set of models, referred to as the regional models, used the 28‐lake data set to compare patterns across lakes with varying characteristics. We then narrowed our focus to examine drivers of interannual and intraseasonal variability in phytoplankton abundance using two different data sets: the first used Green Lakes data collected approximately biweekly from 2008 to 2016 (interannual model), and the second used weekly Loch Vale data from 2015 to 2017 (intraseasonal model).

We implemented all BRT models in the *gbm* package (Ridgeway 2006) of R version 3.5.0 (R Development Core Team, 2018). Chlorophyll *a* concentration, the response variable for all models, was natural log‐transformed to achieve normality. We removed the most highly correlated predictor variables based on Pearson’s coefficients (*r* ≥ |0.8|) and then used the methods described by Bertani et al. (2017) to optimize BRT parameters (Appendix S2). In all described models, we used a backward‐selection procedure to remove variables of low importance iteratively, starting with variable importance (VI) ≤ 1% and ending with VI ≤ 5%, selecting the model that produced the highest cross‐validated coefficient of variation (CV *R* ^2^; Elith et al. 2008). The CV *R* ^2^ is a measure of the fitted models’ ability to predict a subset of observations, and the training *R* ^2^ is a measure of the overall fit to the data set (see Appendix S2 for additional description). We first developed a regional BRT model for the data set that combined environmental, climate, and watershed predictors for all sample lakes, but this resulted in a low CV *R* ^2^ of 0.29. Three separate regional models were subsequently developed for (1) environmental, (2) climate, and (3) watershed predictors (Waite and Metre 2017). Using the backward‐selection procedure described above, we selected the models with the highest CV *R* ^2^ values as the top environmental, climate, and watershed models (Appendix S2: Table S3). Because the CV *R* ^2^ from the regional environmental model was poor, we also created a second combined regional model that included only climate and watershed variables (regional climate + WS), with the rationale that watershed predictors in turn influence water chemistry. Separate BRTs were developed for the Green Lakes Valley (long‐term model) and Loch Vale Watershed (intraseasonal model) data sets. We focused interpretations on variables with ≥5% VI scores because they had the strongest influence on overall model fit (De’ath and Fabricius 2000, Elith et al. 2008). VI is the number of times the variable is used for splitting, weighted by the improvement to the model that is made by including the split.

We explored linear mixed‐effects models (LMMs) as a way to account for correlations among observations that were collected in the same lake or on the same date, but the results did not yield any insight into drivers of chlorophyll *a* in the regional data set (Appendix S3: Table S1). The regional LMM had a very low *R* ^2^_c_ of 0.024 (the variance explained by fixed effects) and moderate *R* ^2^_m_ of 0.451 (the variance explained by fixed and random effects), and none of the fixed effects emerged as significant predictors (all *P* > 0.05). The structure and flexibility of BRT models, combined with their robustness to nonindependent data sets, provided stronger insight into the mechanistic drivers of chlorophyll *a*. Thus, we report only the BRT model results below. All code and data are publicly available; see Data Availability section.

## Results

### Climate and weather

Summers (June–August) during the regional survey years of 2015–2016 were drier and warmer than the 1981–2010 average (6.3 cm/month and 9.2°C mean precipitation and temperature, respectively); 69% of observations occurred when monthly precipitation was ≤50% of the 30‐yr average, and 75% of the observations occurred when monthly air temperature was ≥112% of the 30‐yr average (Appendix S2: Fig. S9). In contrast to summer precipitation, maximum SWE of the preceding winter–spring indicate that 2015–2016 were near or above the 30‐yr averages and ranged from 95 to 125% of normal SWE. However, snow‐free dates were earlier than the long‐term average in this region (Table 1, Appendix S2: Fig. S8).

The long‐term data set spanned a wide range of maximum SWE values and monthly precipitation values Appendix S2: Figs. S8, S9), but all of the driest summer conditions (≤50% of normal) occurred in 2015 and 2016. In the intraseasonal data set, 56% of observations occurred when summer monthly precipitation was <50% of the 30‐yr average and the mean of 76% indicates these summers were drier than normal (Appendix S2: Table S1, Fig. S7). Most of the observations (67%) occurred when the mean summer monthly air temperature was ≥100% of the 30‐yr average (Appendix S2: Table S1, Fig. S7).

### Regional model

**Fig. 2:**
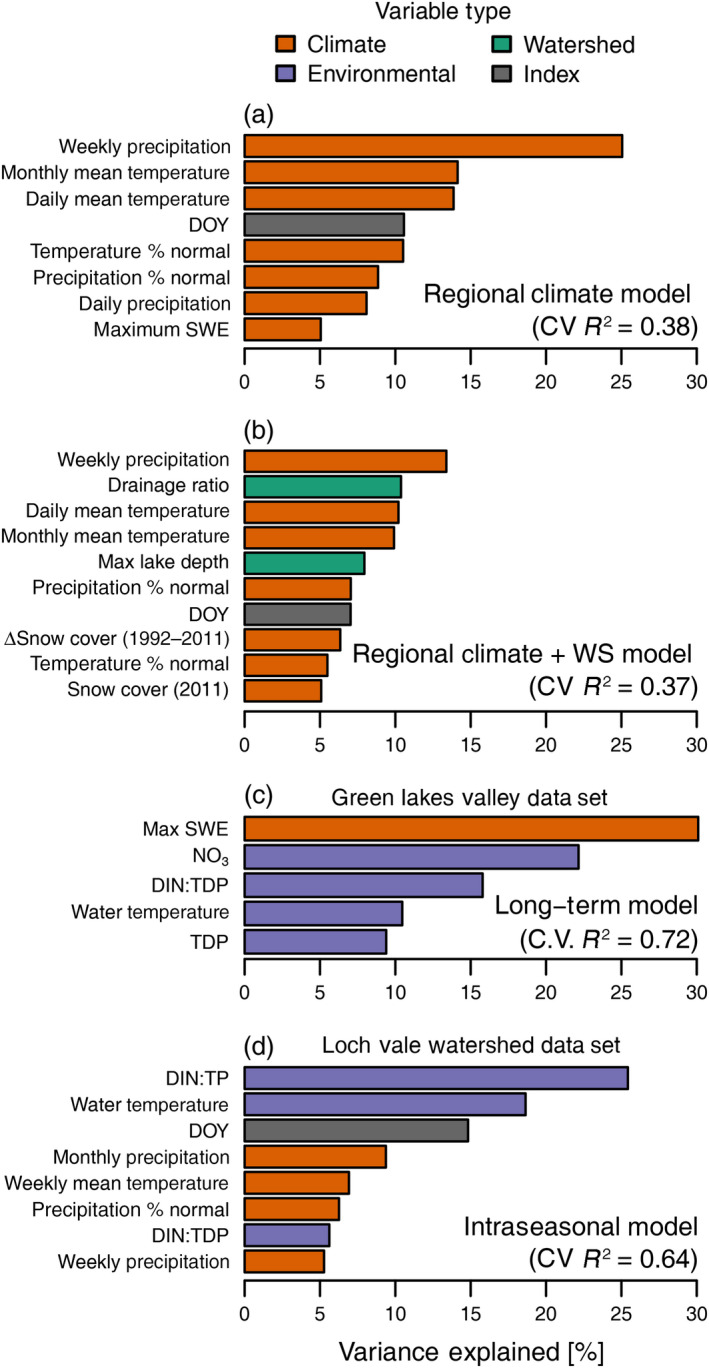
Bar plots listing the top predictor variables (VI > 5%) in (a) the best regional climate, (b) regional climate + watershed (WS), (c) long‐term (Green Lakes Valley), and (d) intraseasonal (Loch Vale watershed) models. The *x*‐axis refers to the percent variance explained by each of the top predictors. Color references to variable type (climate, environment, or index). No watershed predictors emerged as significant predictors in any of the best‐performing models. Refer to Table 1 for predictor variable explanations.

Lake chlorophyll *a* concentrations were variable across regional surveys in 2015–2016 and ranged from highly unproductive to mesotrophic (0.3–23.3 µg/L), with a median of 3.7 µg/L (Table 1, Appendix S2: Fig. S2). All model combinations of predictors performed poorly across the regional survey. Regional models that included all predictor variables, environmental‐only, or watershed‐only variables could not predict lake chlorophyll *a* (Appendix S2: Table S3). The climate‐only model (hereafter regional climate model) was the best‐performing model for regional chlorophyll *a* with a training *R* ^2^ of 0.83 and CV *R* ^2^ of 0.38 (Fig. 2a; Appendix S2: Fig. S1). Influential predictor variables (VI ≥ 5%) included weekly precipitation (VI = 25.1%), monthly mean air temperature (VI = 14.1%), daily mean air temperature (VI = 13.9%), day of year (DOY) of sample collection (VI = 10.6%), monthly air temperature as a percent of 30‐yr normals (VI = 10.5%), monthly precipitation as a percent of 30‐yr normals (VI = 8.6%), daily precipitation (VI = 8.1%), and maximum SWE of the previous winter (VI = 5.1%). Although 2015 and 2016 were average snow years, summer air temperatures were well above normal at all sites (Appendix S2: Figs. S6, S7), with highest chlorophyll concentrations during the driest weeks (Appendix S2: Fig. S3). There were a few exceptions to this finding, where increased chlorophyll concentrations were seen with higher precipitation values in a subset of observations (15%, *n* = 26) during a single week that was wetter than normal.

The combined regional climate + WS model performed similarly to the regional climate model but was less parsimonious (CV *R* ^2^ = 0.37; Fig. 2; Appendix S2: Table S3, Fig. S4). This model identified eight key explanatory variables (VI > 5%); there was some overlap with top predictors in the regional climate model, but the following also emerged as important predictors: lake area as a percentage of watershed area (drainage ratio; VI = 10.3%), maximum lake depth (VI = 7.9%), change in perennial snow and ice cover between 1992 and 2011 (VI = 6.3%), and perennial snow and ice cover (VI = 5.0%).

### Long‐term model

Chlorophyll *a* of lakes in the long‐term data set spanned a similar range as the regional data set, from 0.01 to 19.9 µg/L with a median value of 2.1 µg/L (Appendix S2: Table S1, Fig. S2). Using the long‐term model to explore drivers of interannual variability in lake chlorophyll *a* from 2008 to 2016, maximum observed SWE of the preceding winter (VI = 30.1%) and water column NO_3_ (VI = 22.2%) had the biggest influence on chlorophyll *a* (Fig. 2, Appendix S2: Fig. S5). Dissolved inorganic N to total dissolved P molar ratios (DIN:TDP; VI = 15.8%), water temperature (VI = 10.5%), TDP (VI = 9.4%), and mean monthly air temperature (VI = 4.5%) also influenced chlorophyll *a* values. The long‐term model had a training *R* ^2^ of 0.96 and CV *R* ^2^ of 0.72 (Appendix S2: Fig. S1), and it uncovered three interaction terms (Appendix S2: Fig. S7). The strongest interaction occurred between NO_3_ and maximum SWE, with lower SWE leading to higher NO_3_ and consequently highest predicted chlorophyll *a*. Interactions between low N:P and lake water temperature and earlier snow‐free date also predicted high chlorophyll concentrations (Appendix S2: Fig. S7).

### Intraseasonal model

Chlorophyll *a* in the intraseasonal data set ranged between 0.3 and 11.3 µg/L (Appendix S2: Table S1, Fig. S2). The intraseasonal model had better predictive capabilities than either of the regional models (training *R* ^2^ = 0.93, CV *R* ^2^ = 0.64; Fig. 2, Appendix S2: Fig. S6). Dissolved inorganic N to total P ratios (DIN:TP; VI = 25.4%) and water temperature (VI = 18.6%) were the most important variables, followed by DOY (VI = 14.8%), monthly precipitation (VI = 9.4%), weekly mean temperature (VI = 6.9%), monthly precipitation as a percentage of 30‐yr normal values (VI = 6.3%), dissolved inorganic N to dissolved P molar ratios (VI = 5.6%), and weekly precipitation (VI = 5.3%).

## Discussion

Phytoplankton in southern Rocky Mountain lakes was responsive to both winter and summer precipitation and summer air temperature, though the dominant drivers were dependent on spatial and temporal context. For instance, at interannual timescales, snowpack controlled the magnitude of phytoplankton biomass by regulating nutrient concentrations and water temperature, and summer meteorology explained the most variation across space. Inferring broad‐scale spatial patterns in conjunction with temporal dynamics is often difficult; Lottig et al. (2017) found that drivers of spatial patterns in water clarity could not explain the same temporal dynamics within lakes. Similarly, Leach et al. (2019) found spatial correlations between DOC and TP but no relationship between the two parameters within lakes over time. Both these studies, as well as ours, highlight that the drivers of lake processes at large spatial scales are often fundamentally different from temporal drivers.

When we looked across the 28 lakes in our study, the most important drivers of phytoplankton biomass were summer meteorological conditions, especially weekly precipitation amounts throughout the open‐water season. Snowpack, nutrients, or landscape features did not emerge as the most important drivers of phytoplankton biomass when lakes across the region were compared with each other, contrary to our expectations. Instead, we found that in years with average SWE, summer precipitation extremes and warming enhanced phytoplankton biomass.

In North American lakes located in regions with less topographic complexity, heterogeneity in factors like landscape cover, lake morphometry, and nutrient loading can lead to large variation in responses to the same climatic drivers (Rose et al. 2016, McCullough et al. 2019). In the southern Rocky Mountains, lake locations in small headwater basins with low vegetation cover, short open‐water seasons, and extreme topographic relief seems to simplify the drivers of lake phytoplankton down to weekly precipitation (or lack thereof), monthly and daily temperatures, and a few morphometric characteristics. Although variation in phytoplankton responses to summer weather was high across the region, the size of the lake relative to the watershed, lake depth, and perennial snow and ice cover were important in modulating lake‐to‐lake responses. Specifically, lakes with smaller lake area–to–watershed ratios and deeper lakes generally had higher phytoplankton biomass. Lake‐to‐lake phytoplankton variability in response to summer meteorology was likely high because internal lake processes ameliorate responses to external drivers on different time scales (Baron and Caine 2000).

The modest CV *R* ^2^ of the regional model indicates there are likely missing variables that could predict landscape variation in chlorophyll *a* such as mixed layer depth, stratification, light profiles, and biological community structure. Furthermore, 1–2 samples per lake may not be enough samples to capture the average conditions in a given lake. Variation in watershed and lake morphometry can also drive large differences in algal community structure (Heil et al. 2007, Muylaert et al. 2009), algal traits (Litchman and Klausmeier 2008), and food web structure (Post et al. 2000), all of which influence phytoplankton abundance, but we could not explicitly account for these ecological processes in the models. Furthermore, point estimates of phytoplankton biomass and the land cover predictors are static measures that may not be able to fully integrate spatiotemporal interactions, a limitation that has been pointed out in other macroscale studies of lakes (Lottig et al. 2017).

Climatic patterns emerged at the interannual and seasonal scales, and illustrate the importance of direct and climate‐mediated effects on nutrients at both timescales. Similar to other studies of mountain lake ecosystems, we found that snowpack was the dominant control on interannual variability in lake phytoplankton and nutrient concentrations, with an inverse relationship between chlorophyll *a* and maximum SWE of the previous winter. Snowpack and duration of ice cover influence limnological properties that govern phytoplankton biomass, such as water residence time, stratification, and nutrient concentrations in mountain ecosystems (Thompson et al. 2005, Adrian et al. 2009, Preston et al. 2016, Sadro et al. 2018). In the SRM, high N deposition for over 70 yr (Baron 2006) has led to P limitation of phytoplankton, high N:P in lakes, and generally higher chlorophyll *a* than SRM lakes in lower deposition areas (Elser et al. 2009b). In these lakes, chlorophyll *a* was explained by water temperature and the relative availability of DIN to TP. These, in turn, were most influenced by snowpack, as described by others (Preston et al. 2016), glacier coverage, but also by summer weather patterns (Fig. 2). Nitrate and N:P ratios strongly affected phytoplankton biomass, where high NO_3_ and low N:P ratios were positively related to chlorophyll *a*. Peak seasonal phytoplankton biomass consistently coincided with the warmest water temperatures and lowest N:P ratios within a season.

Like other studies, we found it difficult to infer broad‐scale spatial patterns in conjunction with temporal dynamics (Lottig et al. 2017, Leach et al. 2019). Nonetheless, the contrasting, but complimentary, results from our investigations at regional, seasonal, and interannual scales illuminate various controls on SRM phytoplankton dynamics, which we expand upon below.

### The role of snowpack

In the Sierra Nevada of California, which is characterized by large seasonal snowpack, lower spring SWE leads to warmer lake temperatures, higher nutrient concentrations, and, in turn, enhanced phytoplankton biomass (Sadro et al. 2018a, Sadro et al. 2018b). Similarly, in the southern Rocky Mountains, low spring SWE results in higher summer temperatures and nutrient concentrations (Preston et al. 2016). Our results provide additional support for these mechanistic links between snowpack, nutrient concentrations, water temperature, and phytoplankton biomass as we observed higher chlorophyll *a* with lower maximum SWE across our 8‐yr study period (Fig. 2C). As the season progressed toward baseflow conditions, the relative availability of N to P decreased and temperature increased, and was associated with higher chlorophyll *a* (Appendix S2: Fig. S6). The combination of warmer temperature and lower N:P may have alleviated nutrient and energy limitation, stimulating algal productivity (Cross et al. 2015). Although not supported by data from these lakes prior to 1995 (Baron and Caine 2000), our results, and those of Preston et al. (2016), suggest that lake responses from 2008 to 2016 responded to external influences, in this case winter snowpack.

The interplay between timing of snowmelt, water chemistry, and algal biomass is partially dependent on glaciers and rock glaciers, which are present in both watersheds investigated with the long‐term and intraseasonal models, but not in all watersheds in the data set used for the regional models (Appendix S1: Table S1). Glacial inputs alter the biogeochemistry and phytoplankton ecology of headwater lakes with N‐rich meltwater (Saros et al. 2010). Both glaciers and rock glaciers in the SRM are important sources of NO_3_ to headwater aquatic ecosystems (Baron et al. 2009, Fegel et al. 2016). Nitrate release may result from a combination of microbial nitrification and stored atmospheric N deposition (Slemmons et al. 2013). Glacial‐fed GL4 has significantly higher NO_3_ concentrations than GL1, a snowmelt‐only fed lake; this resulted in strong negative correlations between lake NO_3_ concentrations and increasing snowpack (i.e., dilution) in nonglacial GL1 but not in glacial GL4. Glacier meltwater provides N as well as P, fueling phytoplankton growth in headwater lakes, particularly during dry and warmer‐than‐average summers, like 2015 and 2016. However, even in mountain watersheds without glaciers, low‐snow years can result in increased water column nutrient concentrations because they are not diluted by snowmelt (Park et al. 2004, Parker et al. 2008, Sadro et al. 2018b).

### The role of summer weather

Some of the highest chlorophyll *a* concentrations we observed in the long‐term data set occurred in years that had average snowpack. Several mechanisms could explain why. Although maximum SWE was average in 2015 and 2016, this metric does not capture variability in the timing of snowmelt onset or duration of snowmelt, which can be shortened by warmer, drier summer conditions (Fassnacht et al. 2018), ultimately affecting lake thermal and chemical properties that are important controls on lake productivity (e.g., NO_3_ concentrations, Appendix S2: Fig. S7). Dry summers may also increase the amount of lake evaporation relative to inflow, which concentrates nutrients in the water column (Webster et al. 1996, Lewis et al. 2015) and increases water residence times (Schindler et al. 1996). Our regional model revealed that both the driest and wettest weeks led to high chlorophyll *a* concentrations (Appendix S2: Fig. S4a), suggesting that episodic, convective thunderstorms may have also played a role in increasing phytoplankton biomass by replenishing epilimnetic nutrients through wind‐driven mixing (Sadro and Melack 2012, Perga et al. 2018). Intense storms may additionally decrease water transparency, providing protection to UV‐B–stressed phytoplankton (Sommaruga and Psenner 1997, Parker et al. 2008). Given that summer precipitation represents a relatively minor fraction of annual precipitation budget in the southern Rocky Mountains (Baron and Denning 1993), most likely a combination of both anomalously dry and warm summer conditions resulted in overall higher water temperature and higher nutrient concentrations because of longer residence times and less snowmelt influence, enhancing phytoplankton growth.

Air temperature influences lake temperature and nutrient concentrations either directly via sensible heat flux or indirectly by modifying stratification dynamics (Michelutti et al. 2016). In shallow, mixed lakes, warm air temperatures alone increase water temperatures and stimulate primary production by increasing metabolic rates (Kraemer et al. 2016). Warm temperatures can also concentrate chlorophyll *a* in the upper mixed layer of stratified lakes (Kelly et al. 2018). In other mountainous systems, increased phytoplankton biomass is also a consequence of heat waves (Lepori et al. 2018). As winter snows diminish and summer temperatures continue to warm, the role of summer weather will become more important to mountain lake temperatures, chemistry, and phytoplankton dynamics.

### The importance of watershed context

Although our models do not demonstrate mechanistically how phytoplankton respond to deviations in climate at the regional scale, summer precipitation and air temperature interact with local watershed characteristics and landscape position to regulate the nutrient concentrations that ultimately govern phytoplankton abundance. Land cover can influence the quantity of P delivery to lakes (Wagner et al. 2011), and hydrologic connectivity can influence how much N is processed or exported downstream (Sadro et al. 2012). Landscape position and lake morphometry explain interlake variability in chlorophyll *a*. Chlorophyll *a* in Sky Pond, the larger alpine lake below a glacier deeper, was less directly affected by variations in precipitation compared to The Loch, the shallower downstream subalpine lake due to the moderating influence of cold, glacial meltwater on headwater lakes and the differences in catchment size, as also described by Baron and Caine (2000). Similar contrasts were observed over many years in the Green Lakes Valley, where glacial‐fed GL4 was consistently colder than snow‐fed GL1.

### Conceptualizing cross‐scale drivers of mountain lake phytoplankton

**Fig. 3:**
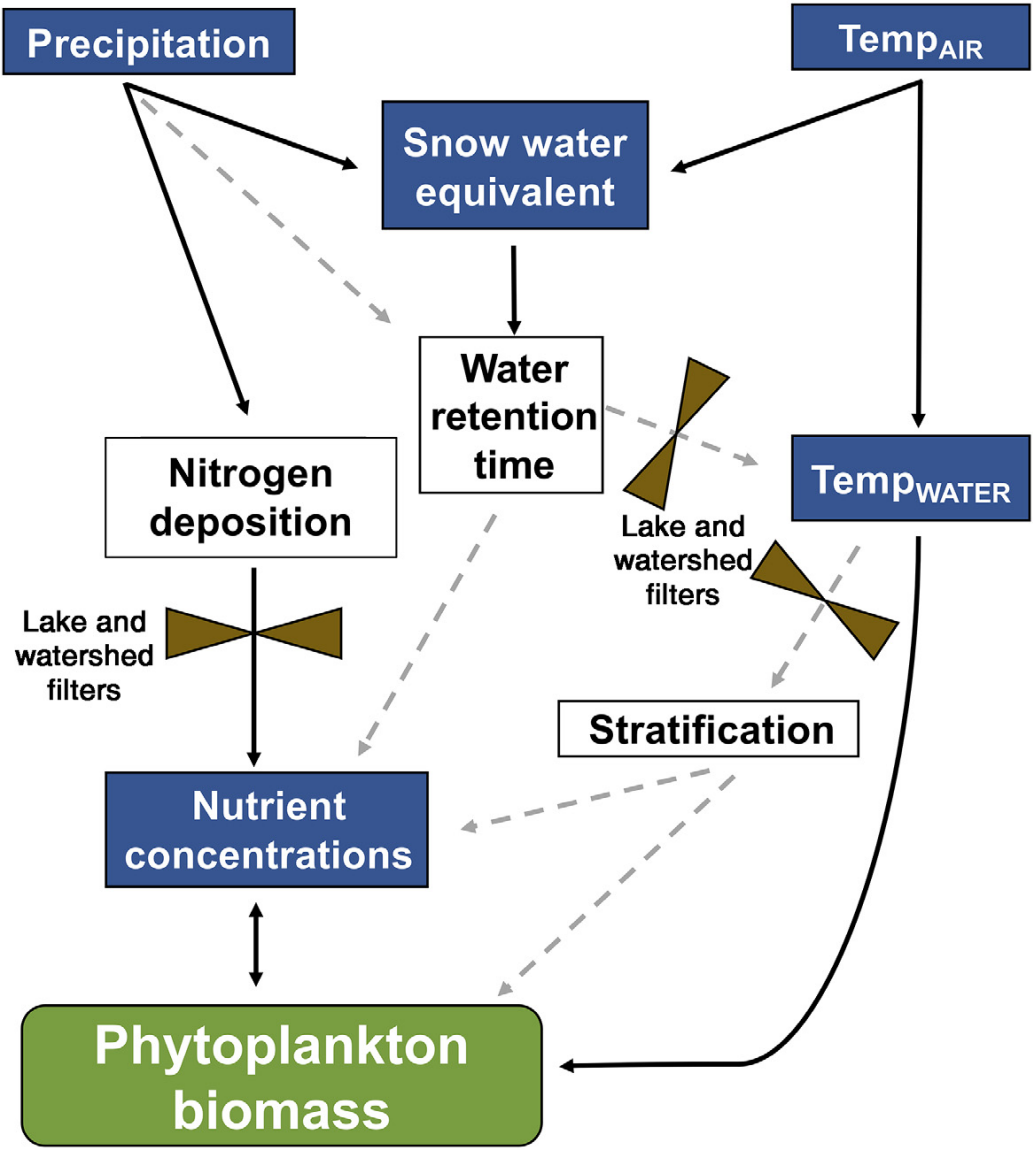
A conceptual framework depicting pathways of physical and chemical drivers of phytoplankton biomass in mountain lakes. Blue boxes represent model‐identified variables significantly influencing lake dynamics and predicting patterns in phytoplankton dynamics. White boxes represent processes not directly measured in our study that are known to influence drivers that influence phytoplankton. Black arrows depict direct relationships; dashed arrows depict indirect relationships. Control valves depict lake‐ or watershed‐specific filters that modify the influence of specific predictors. Precipitation and air temperature have direct and indirect effects on water temperature (Temp_WATER_). Snow water equivalent influences water retention time (e.g., flushing) and nutrient concentrations. Nitrogen deposition influences nutrient concentrations, but lake‐specific concentrations are moderated by lake and watershed filters (land cover, lake morphometry and depth, glaciers), landscape position, and nutrient uptake.

Our combined results allowed us to examine how processes at multiple spatial and temporal scales influence mountain lake phytoplankton. We drew on these results to propose a conceptual framework linking the chemical and thermal limnological properties that give rise to variation in phytoplankton biomass (Fig. 3). In years with summers characterized by anomalously dry and warm weather (2015 and 2016), weekly precipitation and mean monthly air temperatures controlled chlorophyll *a* concentrations, and by inference, lake primary productivity. Dry and warm summer periods enhance evapotranspiration and evaporation, which concentrates nutrients, resulting in higher phytoplankton biomass. Episodic heavy precipitation may also deliver nutrients, colored dissolved organic matter, glacial flour, or other particles that potentially alleviate UV‐B radiation stress and enhance phytoplankton growth. SWE influences lake residence time, with high SWE years typically having high flushing rates and lower nutrient concentrations resulting in lower phytoplankton biomass, but low or average SWE years having less influence than summer weather (Preston et al. 2016, Sadro et al. 2018b). Lake and watershed filters, such as lake depth, catchment position, and presence of perennial ice and snow moderate lake temperatures and nutrients, influencing lake‐specific phytoplankton responses.

Summers in the southern Rocky Mountains have been trending warmer and drier and are changing faster than winter climatic conditions (Fassnacht et al. 2018). Because of this, the influence of summer drought and warmer‐than‐average temperatures will increase in importance in regulating algal growth. We anticipate that continued warming of air and water temperatures in combination with earlier snowmelt and longer ice‐free seasons may lead to increased phytoplankton biomass in high‐elevation lakes (Stewart 2009, Clow 2010, Christianson et al. 2019). Nutrient inputs from atmospheric deposition and the cryosphere coupled with a changing climate could have complex implications for lake stoichiometry and ultimately primary production (Ren et al. 2019). More thoroughly assessing the role of watershed factors in moderating or amplifying lake responses will help us quantify which lakes are more resistant or resilient to environmental change. Our study did not model biological interactions, but future investigators should consider the role of top‐down influences and trophic interactions (McIntire et al. 2007, Ellis et al. 2011).

## Conflict of Interest

None declared.

## Supporting information

Appendix S1

Appendix S2

Appendix S3

## Data Availability

All data and code are available on Zenodo: https://doi.org/10.5281/zenodo.3873194
