# Supplementary material for: The role of warm, dry summers and variation in snowpack on phytoplankton dynamics in mountain lakes
Source: Ecology. 2020 Sep 16;101(10):e03132. doi: 10.1002/ecy.3132 (PMC7583380; doi:10.1002/ecy.3132)
Supplement: Supplementary file 1 — Appendix S1 [file ECY-101-e03132-s001.pdf]

Supporting Information. Oleksy, I.A., W.S. Beck, R.W. Lammers, C.E. Steger, C. Wilson, K. Christianson, K. Vincent, G. Johnson, P.T.J. Johnson, and J.S. Baron. 2020. The role of warm, dry summers and variation in snowpack on phytoplankton dynamics in mountain lakes. Ecology.

Appendix S1 – Chemistry, spatial statistics, and data extraction

All samples were filtered within 24 hours of collection and frozen until analysis. Water temperature and conductivity were measured *in situ* with a hand-held probe (Thermo Scientific Orion 3-Star). Fish presence or absence data were based on investigator site-specific knowledge or through fish stocking records from Colorado Parks and Wildlife. We included sampling depth as a predictor in the models to account for differences in drivers between epilimnion and hypolimnion samples.

With the exception of LVWS lakes, all water samples were analyzed at the Arikaree Environmental Laboratory (Boulder, CO) using standard procedures as described below. Fluorescence spectroscopy was used to determine chlorophyll *a* concentrations (ISA Jobin Yvon-SPEX Fluormax-2 Spectrofluorometer; EPA Method 445.0); reactive nitrogen (NO_3^- and NH_4^+) and TDP concentrations were analyzed with spectrophotometric detection on a Lachat QuickChem 8500 System Flow Injection Analyzer and Alpkem autoanalyzer, respectively (O'Dell, 1993). Loch Vale samples were analyzed according to standard procedures (https://www2.nrel.colostate.edu/projects/lvws/) for the Loch Vale program at the EcoCore laboratory (Fort Collins, CO). Concentrations of chlorophyll *a* were analyzed using a benchtop fluorometer (Turner Designs Trilogy; EPA method 445.0, Arar and Collins 1997). Loch Vale

reactive nitrogen (NO_3^- and NH_4^+) were analyzed on an Alpkem Flow Solution IV Automated wet chemistry system (O.I. Analytical, College Station, TX). Loch Vale TP samples were sent to High Sierra Water Laboratory (Tahoe City, CA) for analysis using persulfate digestion (O'Dell, 1993).

We used the *prism* package (Hart and Bell, 2015) in R version 3.5.0 (R Core Team 2018) to obtain estimates of mean daily, weekly, and monthly temperature and precipitation for each study site from the parameter-elevation regressions on independent slopes model (PRISM Climate Group 2018). For each sample date, we extracted the daily mean temperature and total precipitation and calculated the mean daily temperature and total precipitation for the 7 days and the 30 days preceding the sampling date. To complement these data, we compared monthly temperature and precipitation to climate normal data (1981-2010) for the calendar month closest to the sampling date.

We calculated and extracted several watershed predictors that we hypothesized might play a role in explaining lake-to-lake variation in chlorophyll *a*. Most of these variables did not make it into the final regional, Loch Vale, or Green Lake Valley models, but are reported below. Land cover and recent land cover change for each watershed polygon were estimated using National Land Cover Database (NLCD) data 30 m resolution rasters (Homer et al., 2015). Land cover change from 1992 to 2011 was calculated for forest, wetland, water, and perennial snow and ice by extracting and comparing percent coverage for each year using ArcMap 10.5 Spatial Analyst tools. Additional surficial geology data were extracted from the USGS Geologic Map of North America (Garrity & Soller, 2009). Watershed specific information on rock glacier coverage from the Portland State University Rock Glacier Inventory was added to the land cover database (Johnson, 2018). Slope, aspect, and solar radiation for each watershed were calculated

from the USGS National Elevation Dataset ⅓ arc-second (≈ 10 m) resolution raster and extracted using ArcMap 10.5 Spatial Analyst tools (ESRI, 2018; Nussear et al., 2009). Eastness is derived from degree aspect using the equation: eastness = sin((degree aspect * π)/180); northness is derived from degree aspect using the equation: northness = cos((degree aspect * π)/180). Solar radiation was calculated in watt hours per square meter (WH m_{-2}) at a daily time step, then aggregated to monthly, using unique centroid latitudes for each watershed. A generally clear sky with a transmissivity of 0.5 and diffuse proportion of 0.3 was assumed, with 32 unique horizon calculations considered each hour. Each watershed polygon was buffered by 50 m to include the influences of all immediately surrounding topography in the high and rugged alpine terrain of the study area. We initially attempted to incorporate spatial estimates of atmospheric N deposition in our models; however, these estimates combined data on N deposition and precipitation at different temporal and spatial scales, leading to high levels of uncertainty. Due to these difficulties, we did not include these estimates in the models.

Table S1. Additional summary information for predictor variables used in the Regional models (2015-2016). Summer statistics include minimum, maximum, mean, median, and standard deviation for each predictor variable. Randomly selected monthly observations from Loch Vale watershed and Green Lakes Valley lakes are included in the model and data summary presented below. Dashes indicate data were unavailable or summary statistics could not be computed on categorical variables.

		**Full dataset summary statistics**				
**Watershed variables**	**Description [units]**	**min.**	**max.**	**mean**	**med.**	**std. dev.**
Barren (2011)	Barren land cover [%]	0.0%	96%	45%	41%	21%
Forest (2011)	Forest land cover [%]	0%	92%	8%	1%	15%
Shrub (2011)	Shrub land cover [%]	0%	50%	14%	11%	12%
Snow (2011)	Perennial snow and ice land cover [%]	0%	77%	31%	32%	18%
Wetland (2011)	Wetland land cover [%]	0.0%	1.2%	0.2%	0.0%	0.3%
Change shrub (‘92-’11)	Change in shrub land cover 1999 to 2011 [%]	-1.9%	1.7%	0.2%	0.0%	0.5%
Change forest (‘92-’11)	Change in forest land cover from 1992 to 2011 [%]	0.0%	0.2%	0.0%	0.0%	0.1%
Change snow (‘92-’11)	Change in perennial snow & ice land cover 1992 to 2011 [%]	-3.5%	0.0%	-1.1%	-0.9%	0.9%
Change water (‘92-’11)	Change in water land cover 1992 to 2011 [%]	-0.1%	1.9%	0.1%	0.0%	0.3%
Change wetland (‘92-’11)	Change in wetland land cover 1992 to 2011 [%]	0.0%	0.0%	0.0%	0.0%	0.0%
Biotite-gneiss	Biotite-gneiss underlying geology [%]	0%	100%	51%	56%	36%
Granite	Granite underlying geology [%]	0%	100%	32%	17%	34%
East mean	East-ness of watershed [sin(aspect)]	-0.078	0.138	0.023	0.015	0.038
North mean	North-ness of watershed [cos(aspect)]	-0.049	0.118	0.012	0.012	0.0
Elevation	Elevation at lake outflow [m]	2987	3550	3310	3322	161
Elevation range	Elevation range of watershed [m]	116.0	1317.1	614.7	620.7	228.2
WS area	Watershed area [km^2]	0.2	13.1	3.1	2.2	3.1
Lake SA	Lake surface area [m^2]	3209	163,801	54,691	41,311	39,661
Drainage ratio	Lake area as a percentage of watershed area [%]	0.5%	10.2%	3.1%	2.1%	2.7%
Summer radiation	Total monthly solar radiation [W m^{-2}]	171,494	227,293	202,013	207,416	15,035
Rock glacier area	Rock glacier area [km^2]	0.00	0.56	0.07	0.03	0.09
Rock glacier %	Percent of catchment occupied by rock glaciers [%]	0.0%	19.0%	2.9%	1.5%	3.8%
Longitude	Longitude [decimal degrees]	-105.7	-105.59	-	-	-

Literature cited

Arar, E. J., & Collins, G. B. (1997). Method 445.0: In vitro determination of chlorophyll a and pheophytin a in marine and freshwater algae by fluorescence. Washington, DC, USA: *United States Environmental Protection Agency, Office of Research and Development, National Exposure Research Laboratory.*

Garrity, C. P., & Soller, D. R. (2009). Database of the Geologic Map of North America- Adapted from the Map by JC Reed, Jr. and others (2005). *U.S. Geologic Survey*. Retrieved from https://pubs.usgs.gov/ds/424/

Hart, E. M., & Bell, K. (2015). prism: Download data from the Oregon prism project. R package version 0.0.6. http://doi.org/10.5281/zenodo.33663

Homer, C. G., Dewitz, J. A., Yang, L., Jin, S., Danielson, P., Xian, G., Megown, K. (2015). Completion of the 2011 National Land Cover Database for the conterminous United States-Representing a decade of land cover change information. *Photogrammetric Engineering and Remote Sensing*. http://doi.org/10.14358/PERS.81.5.345

Johnson, G. F. (2018). *Rock Glaciers of the Contiguous United States: Spatial Distribution, Cryospheric Context, and Riparian Vegetation*.

Nussear, K. E., Esque, T. C., Inman, R. D., Gass, L., Thomas, K. A., Wallace, C. S. A.,

O'Dell, J. W. (1993). Method 365.1, Revision 2.0: Determination of Phosphorus by Semi-Automated Colorimetry. In *U.S. Environmental Protection Agency, Washington, DC*.

PRISM Climate Group. (2018). *Oregon State University*. Retrieved from http://prism.oregonstate.edu

R Core Team (2018). R: A Language and Environment for Statistical Computing, Vienna,

Austria. Available at: https://www.R-project.org/.

Webb, R. H. (2009). Modeling Habitat of the Desert Tortoise (Gopherus agassizii) in the Mojave and Parts of the Sonoran Deserts of California, Nevada, Utah, and Arizona. Open-File Report. Retrieved from http://pubs.er.usgs.gov/publication/ofr20091102
