# Supplementary material for: The role of warm, dry summers and variation in snowpack on phytoplankton dynamics in mountain lakes
Source: Ecology. 2020 Sep 16;101(10):e03132. doi: 10.1002/ecy.3132 (PMC7583380; doi:10.1002/ecy.3132)
Supplement: Supplementary file 2 — Appendix S2 [file ECY-101-e03132-s002.pdf]

Supporting Information. Oleksy, I.A., W.S. Beck, R.W. Lammers, C.E. Steger, C. Wilson, K. Christianson, K. Vincent, G. Johnson, P.T. Johnson, and J.S. Baron. 2020. The role of warm, dry summers and variation in snowpack on phytoplankton dynamics in mountain lakes. Ecology.

Appendix S2 – Boosted regression tree methodological description, summary statistics, model results, and visualizations

---

BRTs are used in a variety of aquatic ecology contexts including predicting cyanobacteria bloom occurrence (Bertani et al. 2017), macroinvertebrate biotic integrity (Waite and Van Metre, 2017), marine fish and coral diversity and abundance (Pittman et al. 2009), and littoral fish abundance (Lewin et al. 2014). We used the methods described by Bertani et al. (2017) to optimize the learning rate (contribution of each tree added to the model) and bag fraction (proportion of data randomly drawn at each step). We set the tree complexity (number of splits per tree) to 2, and controlled for over-fitting by ensuring the model used a small learning rate, which requires significant computation time but results in a final model with a larger number of trees (Friedman, 2001; Hastie et al., 2001; Elith et al., 2008). Optimization was based on minimizing the out-of-sample predictive error estimated through 10-fold cross validation. Once we selected the optimal values for learning rate and bag fraction, we used 10-fold cross validation (C.V.) again to determine the optimal number of trees for each model. We evaluated model performance using the C.V. R^2, which is a more conservative evaluation measure than the overall R^2. For each of these models, we used a backward-selection procedure to iteratively

remove variables of low importance starting with V.I. ≤ 1% and ending with V.I. ≤ 5% and selected the models with the highest C.V. R^2 values in each category (Elith et al. 2008).

We assessed individual predictor importance using variable importance (V.I.) scores, which indicate the percentage of trees splits that can be attributed to a single predictor variable. We assessed the importance of interactions between predictor variables using relative interaction strengths, which are calculated in the *gbm* package from the residual variances of linear models that use pairs of all the predictor variables to make predictions.

Elith, J., Leathwick, J. R., & Hastie, T. (2008). A working guide to boosted regression trees. *J Anim. Ecol.*, *77*(4), 802–813.

Friedman, J. H. (2001). Greedy function approximation: a gradient boosting machine. *Annals of Statistics*, 1189-1232.

Garrity, C. P., & Soller, D. R. (2009). Database of the Geologic Map of North America- Adapted from the Map by JC Reed, Jr. and others (2005). *U.S. Geologic Survey*. Retrieved from https://pubs.usgs.gov/ds/424/

Hastie, T., Tibshirani, R., & Friedman, J. (2001). Data mining, inference, and prediction. *The elements of statistical learning Springer Series in Statistics. Springer-Verlag, New York.*

Homer, C. G., Dewitz, J. A., Yang, L., Jin, S., Danielson, P., Xian, G., Megown, K. (2015). Completion of the 2011 National Land Cover Database for the conterminous United States-Representing a decade of land cover change information. *Photogrammetric Engineering and Remote Sensing*. *81*(5), 345-354.

Johnson, G. F. (2018). *Rock Glaciers of the Contiguous United States: Spatial Distribution, Cryospheric Context, and Riparian Vegetation.*

Nussear, K. E., Esque, T. C., Inman, R. D., Gass, L., Thomas, K. A., Wallace, C. S. A.,

Webb, R. H. (2009). Modeling Habitat of the Desert Tortoise (Gopherus agassizii) in the Mojave and Parts of the Sonoran Deserts of California, Nevada, Utah, and Arizona. Open-File Report. Retrieved from http://pubs.er.usgs.gov/publication/ofr20091102

O'Dell, J. W. (1993). Method 365.1, Revision 2.0: Determination of Phosphorus by Semi-Automated Colorimetry. In *U.S. Environmental Protection Agency, Washington, DC*.

Table S1. Summary of all predictor variables used to model chl *a* in the Loch Vale and Green Lakes Valley datasets. Means and standard deviation for watershed variables are not presented because model only included two lakes.

		Loch Vale dataset				Green Lakes Valley Dataset			
**Variable**	**Description [units]**	**min**	**max**	**mean**	**sd**	**min**	**max**	**mean**	**sd**
Response									
Chl *a*	Chlorophyll a [μg L^{-1}]	0.3	11.3	3.7	3.1	0.01	19.9	5.0	5.0
Indexing									
DOY	Ordinal date	152	232	204.3	26.9	189	238	213.6	13.1
Measurement depth	Surface (0) or hypolimnion (1) measurement	0	1	-	-	0	1	-	-
Climate									
Daily precip.	Precipitation on sample date [mm]	0.0	4.9	0.5	1.1	0	16.1	2.3	4.2
Weekly precip.	Cumulative precip. for the week preceding sample date [mm]	0	17.0	6.4	5.3	0	57.2	16.3	15.5
Monthly precip.	Cumulative precip. for the 30 days preceding sample date [mm]	8.8	114.9	42.9	35.5	-	-	-	-
Precip. % normal	Monthly precip. as a percent of normal [%]	24%	184%	76%	40%	30%	148%	97%	38%
Daily mean temp.	Mean air temperature sample date [ºC]	5.6	14.2	11.0	2.7	3.7	13.2	9.7	2.3
Weekly mean temp.	Mean air temperature for the week preceding sample date [ºC]	3.7	14.5	11.2	3.2	-	-	-	-
Monthly mean temp.	Mean air temperature for the 30 days preceding sample date [ºC]	2.4	13.1	10.5	3.5	8.1	11.8	9.8	0.8
Mean temp. % normal	Monthly average air temperature as a percent of normal [%]	86%	150%	118%	20%	92%	152%	107%	12%
SWE % normal	Snow water equivalent (SWE) as a percentage of normal maximum SWE [%]	95%	125%	103%	13%	93%	155%	119%	20%
Max. SWE	Maximum observed SWE for the preceding winter [in]	17.7	18.0	17.9	0.1	19.6	28.4	23.2	3.3
Difference snow free date	Difference between normal and actual snow free date [days]	-2	-1	-1.4	0.5	-6	6	-1.3	4.8
Environmental									
DOC	Dissolved organic carbon [mg L^{-1}]	0.3	2.8	0.9	0.9	0.4	2.5	0.9	0.4
FISH	Fish absence (0) or presence (1)	1	1			0	1		
Max. lake depth	Maximum lake depth [m]	5	7.2	5.8	1.1	7	13.1	10.6	3.0
NO3	Nitrate-N [mg L^{-1} N]	0.08	0.35	0.2	0.1	0.0	0.2	0.1	0.1
DIN:TDP	Total dissolved N to total dissolved P molar ratio	44.2	686.4	228.8	160.5	53.6	1685.1	328.2	345.8
DIN:TP	Total dissolved N to total P molar ratio	22.3	125.7	57.7	27.2	-	-	-	-
TDN	Total dissolved N [mg L^{-1}]	0.1	0.5	0.2	0.1	0.0	0.4	0.2	0.1

TDP	Total dissolved phosphorus [μg L^{-1}]	0.64	14.4	5.1	3.8	0.3	4.8	1.9	1.1
TP	Total phosphorus [μg L^{-1}]	6	21	10.4	2.6				
Lake temp.	Water temperature of sample [ºC]	3.2	13.9	9.1	2.7	5.8	13.3	10.0	1.6
Watershed									
Barren cover (2011)	Barren land cover [%]	70%	81%	-	-	31.7%	60.4%	-	-
Forest cover (2011)	Forest land cover [%]	0%	8%	-	-	0.0%	0.0%	-	-
Shrub cover (2011)	Shrub land cover [%]	1.4%	6.4%	-	-	7%	50%	-	-
Snow cover (2011)	Perennial snow and ice land cover [%]	14%	15%	-	-	12%	32%	-	-
Wetland cover (2011)	Wetland land cover [%]	0.0%	0.2%	-	-	0.0%	0.3%	-	-
Change shrub ('92-'11)	Change in forest land cover from 1992 to 2011 [%]	0.0%	0.2%	-	-	0%	0%	-	-
Change forest ('92-'11)	Change in shrub land cover 1999 to 2011 [%]	-0.07%	0.00%	-	-	0.0%	0.2%	-	-
Change snow ('92-'11)	Change in perennial snow & ice land cover 1992 to 2011 [%]	-0.9%	-0.6%	-	-	-2.5%	-1.8%	-	-
Change water ('92-'11)	Change in water land cover 1992 to 2011 [%]	0	0	-	-	0.00%	0.16%	-	-
Change wetland ('92-'11)	Change in wetland land cover 1992 to 2011 [%]	0.0%	0.0%	-	-	0%	0%	-	-
Biotite-gneiss	Biotite-gneiss underlying geology [%]	85%	93%	-	-	56%	70%	-	-
Granite	Granite underlying geology [%]	7%	15%	-	-	15%	30%	-	-
East mean	East-ness of watershed	0.016	0.024	-	-	0.008	0.073	-	-
Elevation	Elevation at lake outflow [m]	3048	3322	-	-	3425	3561	-	-
Elevation range	Elevation range of watershed [m]	696.8	902.5	-	-	280.7	520.7	-	-
WS area	Watershed area [km^2]	2.2	6.8	-	-	0.4	2.2	-	-
Lake SA	Lake surface area [m^2]	41126	52731.4	-	-	40016.7	41311.8	-	-
Drainage ratio	Lake area as a percentage of watershed area [%]	0.8%	1.9%	-	-	1.9%	9.8%	-	-
Summer radiation	Total monthly solar radiation [W m^{-2}]	171494	180812	-	-	205694	227294	-	-
Rock glacier area	Rock glacier area [km^2]	0.15	0.19	-	-	0.000	0.035	-	-
Rock glacier %	Percent of catchment occupied by rock glaciers [%]	3%	7%	-	-	0.0%	1.6%	-	-

Table S2. Summary of lake morphometry, watershed (WS) area, and chlorophyll *a*, conductivity, and secchi depth for lakes included in the regional dataset. Water clarity and chemical parameters are reported as the mean of n sampling trips. Missing values are indicated with a dash.

Lake Name	Longitude	Latitude	Elevation (m)	Max. Depth (m)	Lake as % WS area	Lake area (ha)	WS area (km^2)	Water temp. (ºC)	Chl *a* (µg L^{-1})	Secchi (m)	Cond. (µS cm^{-1})	n
Albion	40.0468	-105.6036	3345	15.0	2.4	13.1	5.4	11.6	11.7	3.9	24.2	4
Black	40.2653	-105.6413	3236	31.0	0.6	3.2	5.4	10.0	4.6	5.8	-	2
Blue	40.0889	-105.6198	3449	42.0	3.5	9.2	2.7	6.9	2.4	4.6	5.0	3
Blue 2	40.2679	-105.6316	3408	9.1	4.7	1.0	0.2	17.0	5.2	2.3	-	2
Cony	40.1729	-105.6580	3508	19.2	5.6	5.3	0.9	7.9	1.9	5.0	-	2
Diamond	39.9927	-105.6504	3340	7.0	4.0	5.8	1.4	12.1	2.4	5.3	18.0	3
Finch	40.1834	-105.5930	3021	3.2	10.3	2.4	0.2	13.7	2.8	-	-	2
Forest	39.9212	-105.6737	3307	3.0	2.1	2.2	1.0	8.3	7.9	2.7	16.0	2
Frozen	40.2577	-105.6427	3529	27.0	3.5	2.7	0.8	8.9	4.9	4.9	-	2
GL1	40.0509	-105.6056	3425	7.0	9.8	4.0	0.4	13.2	1.9	7.0	49.2	2
GL4	40.0553	-105.6203	3550	13.0	1.9	4.1	2.2	10.8	3.1	4.5	14.8	2
Haiyaha	40.3046	-105.6622	3118	9.0	1.6	4.6	2.9	11.8	10.0	-	12.4	1
Isabelle	40.0693	-105.6185	3301	8.0	2.7	12.7	4.7	7.1	4.7	5.3	10.0	3
Jasper	39.9790	-105.6628	3301	10.0	2.9	7.6	2.6	6.1	2.8	3.5	11.5	2
Lion 1	40.2319	-105.6386	3373	1.8	0.7	2.0	3.0	11.8	0.6	-	-	2
Lion 2	40.2376	-105.6417	3478	11.4	0.8	1.5	1.8	10.6	4.2	5.0	6.3	5
Long	40.0723	-105.5923	3253	7.0	1.3	16.4	13.1	9.9	3.3	3.1	12.0	3
Lost	39.9496	-105.6165	2987	4.0	10.0	2.0	0.2	13.7	5.0	3.3	39.0	3
Mills	40.2895	-105.6416	3030	8.4	0.5	5.8	12.1	17.0	1.4	3.5	-	2
Pear	40.1767	-105.6267	3226	16.0	3.5	6.5	1.8	12.7	6.4	4.0	8.3	5
Red Deer	40.1427	-105.6101	3163	20.0	6.1	5.9	1.0	12.3	3.9	5.3	10.0	3
Sky	40.2781	-105.6683	3322	7.2	1.9	4.1	2.2	7.8	5.6	3.0	10.0	4
Snowbank	40.2402	-105.6452	3512	8.5	1.8	2.9	1.6	10.0	2.9	4.5	7.3	5
The Loch	40.2926	-105.6562	3048	5.0	0.8	5.3	6.8	10.0	2.1	4.4	13.1	5
Thunder	40.2222	-105.6472	3225	16.2	2.0	6.0	3.0	10.7	5.5	3.3	-	2
Upper Diamond	39.9882	-105.6612	3463	3.5	1.0	0.3	0.3	6.3	1.8	3.2	18.3	3
Upper Hutchenson	40.1738	-105.6477	3412	3.6	1.0	2.6	2.7	9.3	2.0	-	-	2
Yankee Doodle	39.9375	-105.6538	3261	7.5	4.0	1.4	0.3	12.1	4.8	3.0	10.5	2

Table S3. Top predictors from the best performing: (1) environmental, (2) climate, and (3) watershed regional models. Top predictors were produced from a backward-selection process, whereby variables of low importance were removed until cross-validation (C.V.) R^2 values were maximized. Since the WS-only and Envt-only models had lower C.V. R^2 values than the Regional Climate and Regional Climate + WS models, we did not focus our discussion on interpreting these model results.

**Climate-only variables** C.V. $R^2 = 0.38$	**V.I.** (%)	**WS-only variables** C.V. $R^2 = 0.31$	**V.I.** (%)	**Envt-only variables** C.V. $R^2 = 0.15$	**V.I.** (%)
Weekly precip.	25.1	DOY	22.8	DIN:TDP	22.7
Monthly mean temp.	14.1	Max. lake depth	12.6	TDP	19.2
Daily mean temp.	13.8	Drainage ratio	8.5	NO3	17.2
DOY	10.5	Elevation	7.9	Lake temp.	15.7
Temp. % normal	10.5	Change in snow cover ('92-'11)	6.5	TDN	8.0
Precip. % normal	8.6	Shrub cover (2011)	5.7	DOC	6.9
Daily precip.	8.1	Lake SA	5.3	DOY	6.7
Max. SWE	5.1	Snow cover (2011)	5.0	Measurement depth	3.7
		Summer radiation	4.8		
		Biotite-gneiss (%)	4.6		
		East mean	3.8		
		Measurement depth	3.5		
		Rock glacier area	3.3		
		Change in water cover ('92-'11)	3.0		

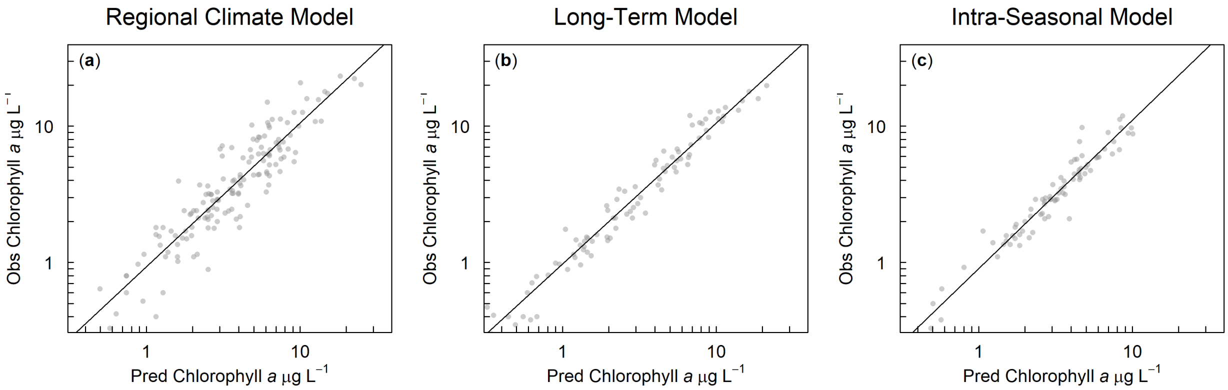

Figure S1. Chlorophyll *a* observed versus predicted values for the best Regional (Climate) (a), Long-term (b), and Intra-seasonal (c) BRT models, with fitted regression lines described by the training R^2 values. Regional Climate model training $R^2 = 0.83$ and C.V. $R^2= 0.38$, Long-term model training $R^2 = 0.96$ and C.V. $R^2 = 0.72$, Intra-seasonal model training $R^2 = 0.93$ and C.V. $R^2= 0.64$.

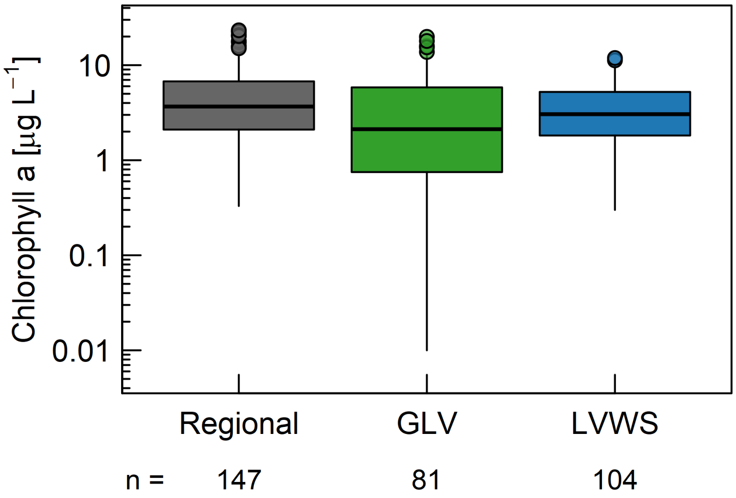

Figure S2. Boxplot of measured chlorophyll a from each of the three datasets. The median of the Green Lakes dataset is significantly less than the Regional dataset, but no other differences are statistically significant. ($p = 0.0016$; Wilcoxon Rank Sum test with Bonferroni correction)

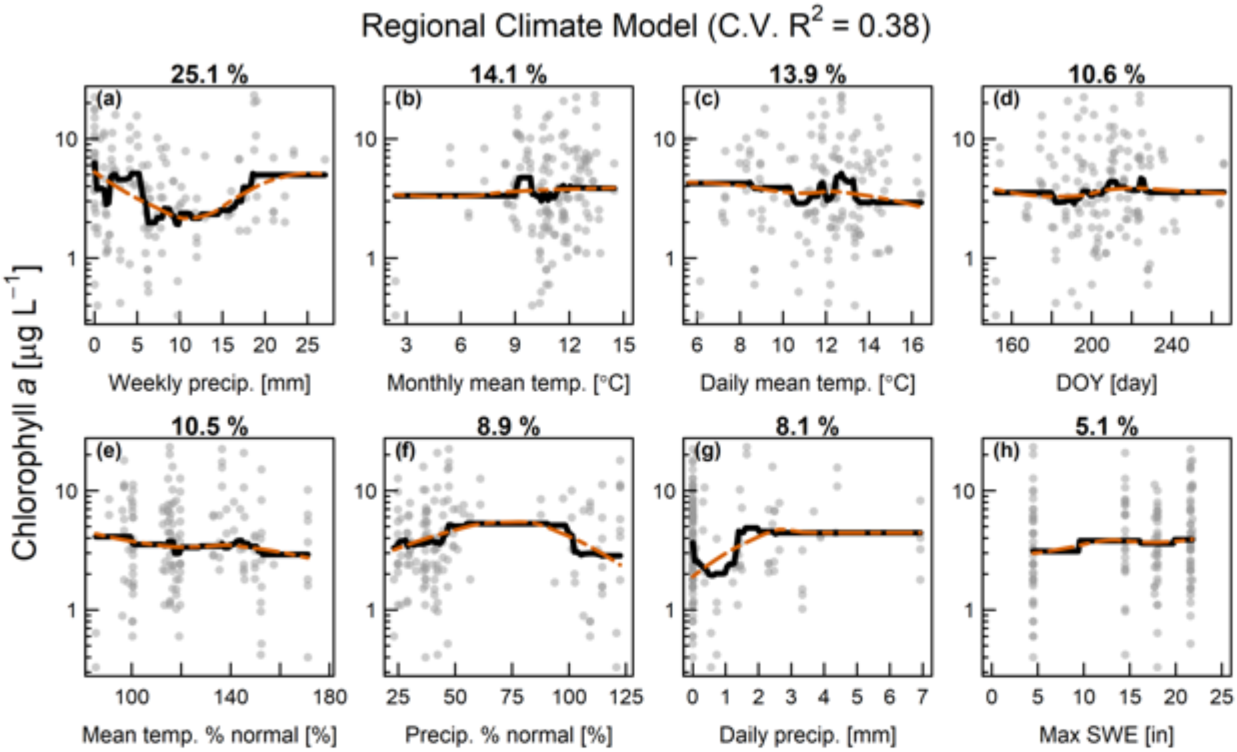

Figure S3. Partial dependency plots of the predictor variables (V.I. > 5%) in the best Regional BRT model including only climate predictors (variable importance ≥ 5%) with the relative

contribution of each variable printed at the top of each panel. The y-axis fitted function (black line) represents the effect of the selected variable on chl *a* when all other predictor variables are held at their mean values. The red dashed line is a loess curve fit to this relationship. Grey dots represent individual observations. Refer to Table 1 for predictor variable explanations.

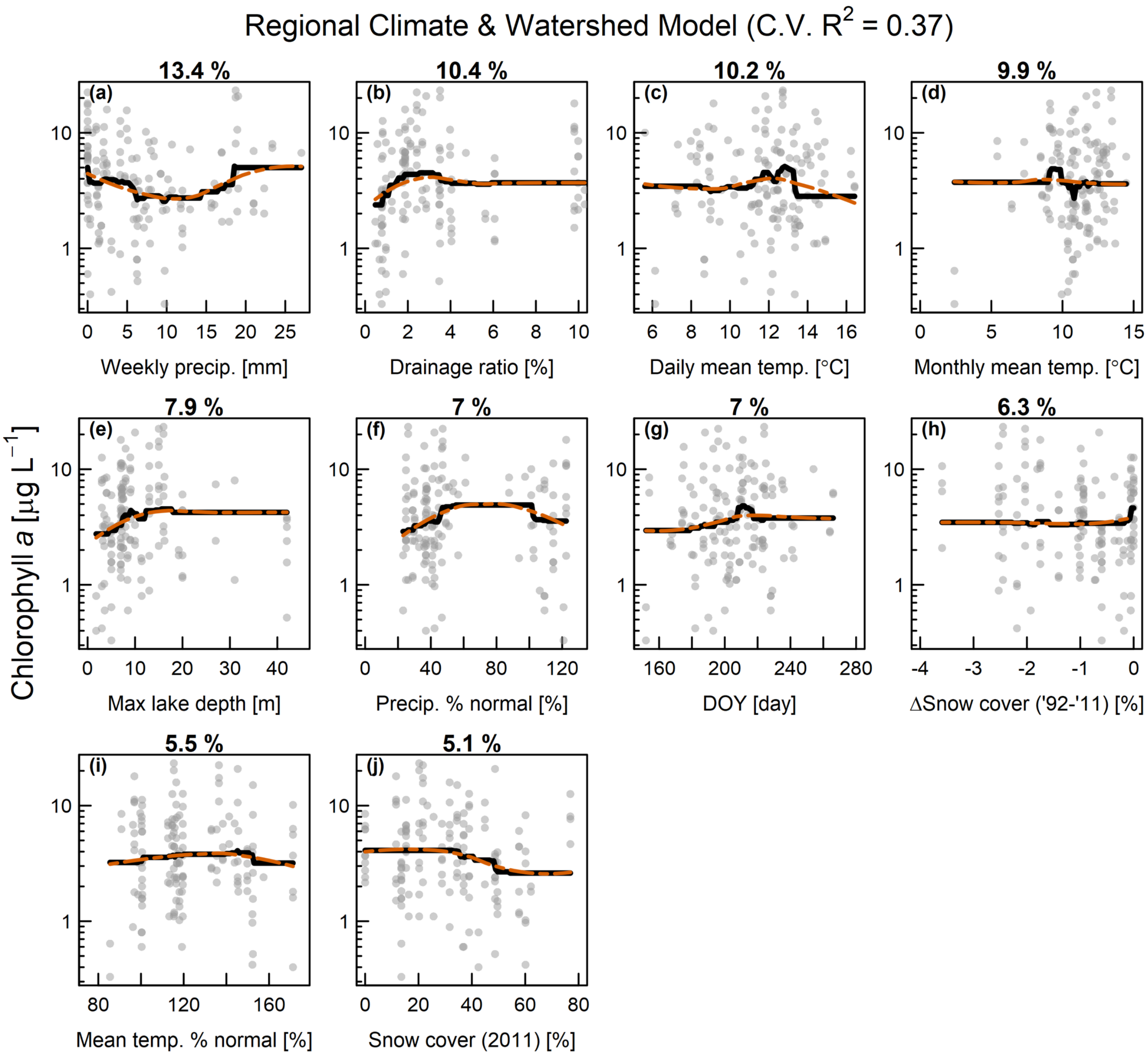

Figure S4. Partial dependency plots of the predictor variables in boosted regression tree analysis from the best regional model with climate & watershed predictors (variable importance ≥ 5%) with the relative contribution of each variable printed at the top of each panel. The y-axis fitted function (black line) represents the effect of the selected variable on chl *a*, holding all other

predictors at their mean value. The red dashed line is a loess curve fit to this relationship. Grey dots represent individual observations. Refer to table 1 and table S1 for predictor variable explanations.

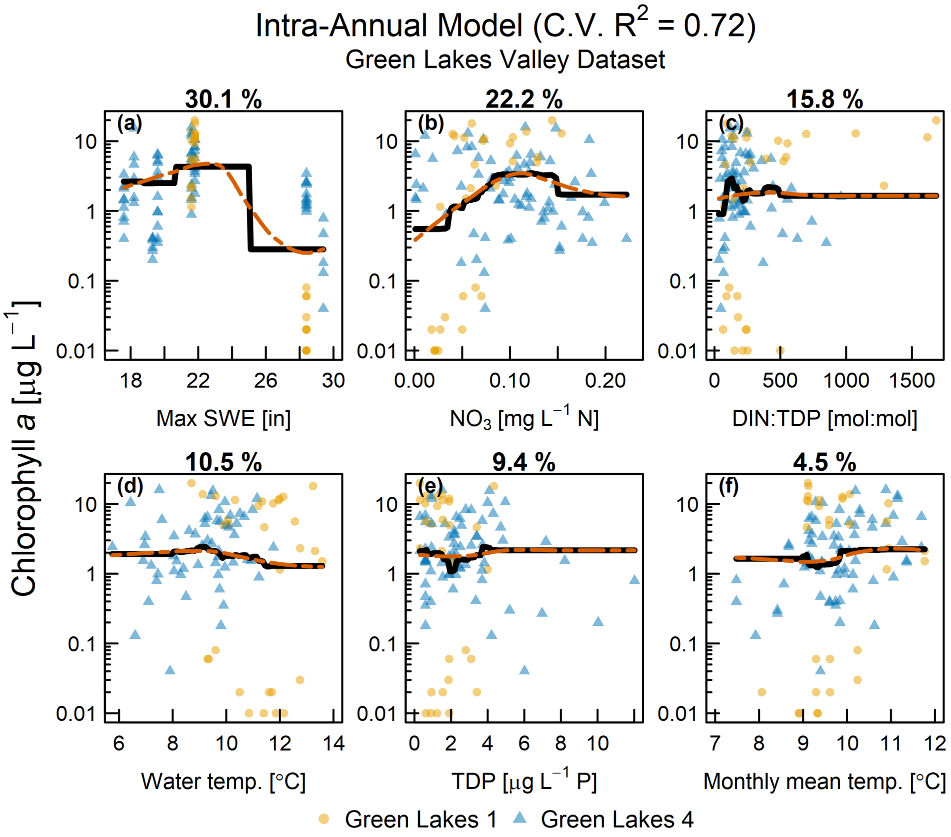

Figure S5. Partial dependency plots for the best Long-term (Green Lakes Valley) model for all variables (V.I. ≥ 5%) with the relative contribution of each variable printed at the top of each panel. The y-axis fitted function (black line) represents the effect of the selected variable on chl *a* with all other predictors held at their means. The red dashed line is a loess curve fit to this relationship. Yellow dots and blue triangles represent individual observations for GL1 and GL4 (respectively). Refer to Table 1 for predictor variable explanations.

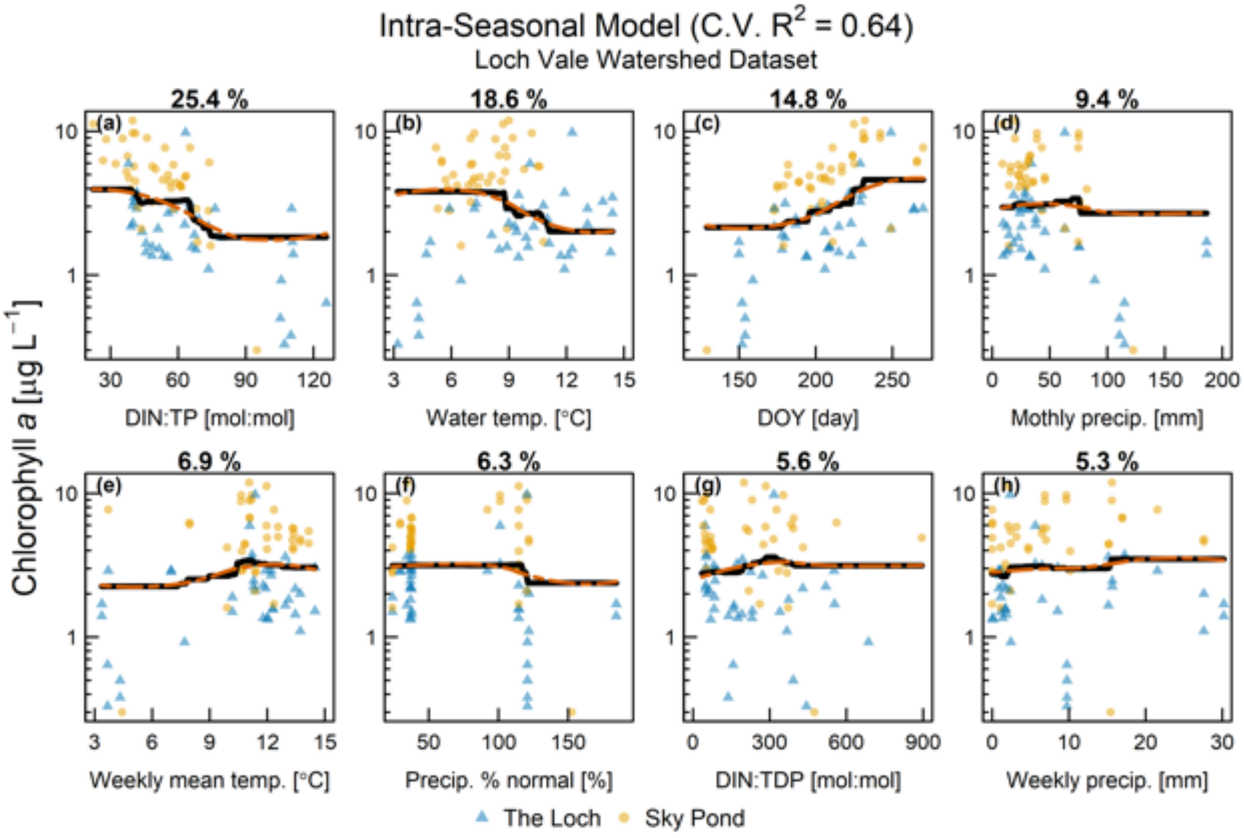

Figure S6. Partial dependency plots for the best Intra-seasonal (Loch Vale watershed) model for all variables (V.I. ≥ 5%). The relative contribution of each variable is printed at the top of the panel. The y-axis fitted function (black line) represents the effect of the selected variable on chl *a*, holding all other predictors at their means. The red dashed line is a loess curve fit to this relationship. Blue triangles and yellow dots represent individual observations for The Loch and Sky Pond (respectively).

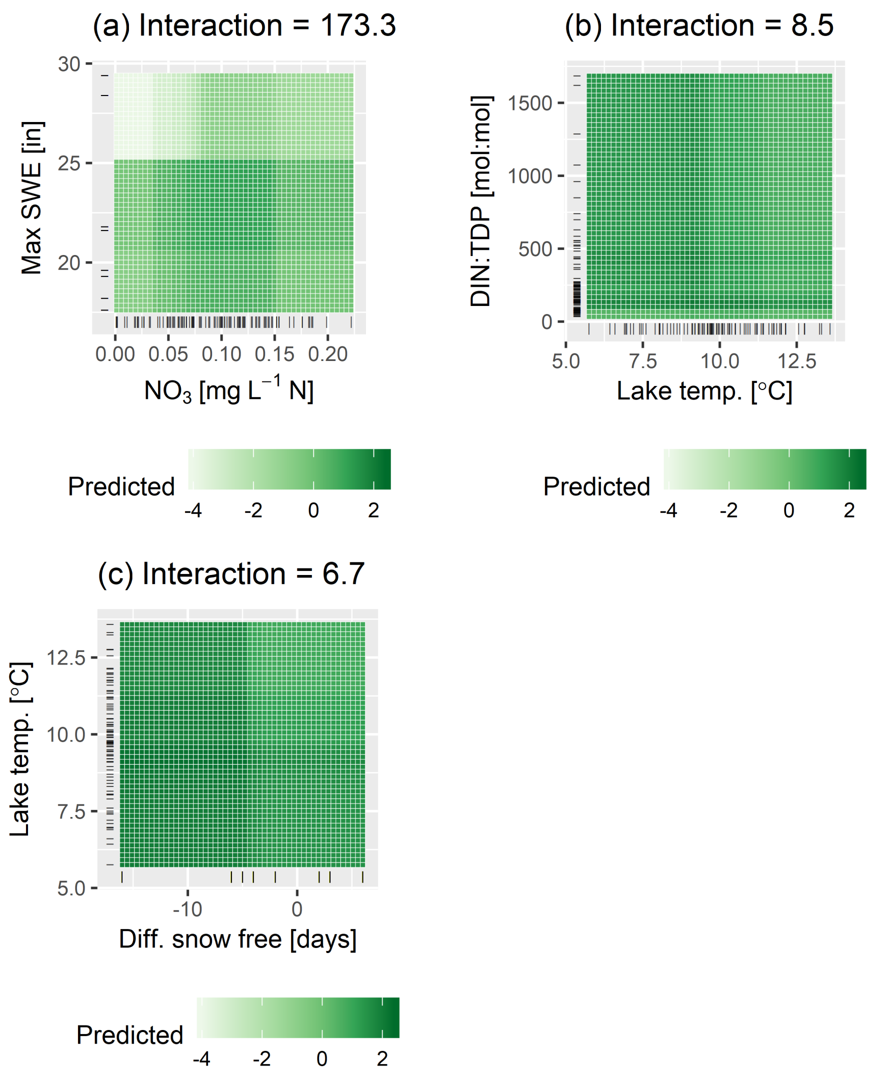

Figure S7. Interaction plots from the best GLV model, ranked by interaction strength: (a) maximum SWE and nitrate, (b) DIN:TDP and lake water temperature, and (c) lake water temperature and difference in snow free days, where negative numbers indicate earlier than normal snowmelt occurrence. Colors are predicted log-chlorophyll *a* with darker shades corresponding to higher concentrations. Hash marks along each axis indicate values of each variable from the GLV dataset.

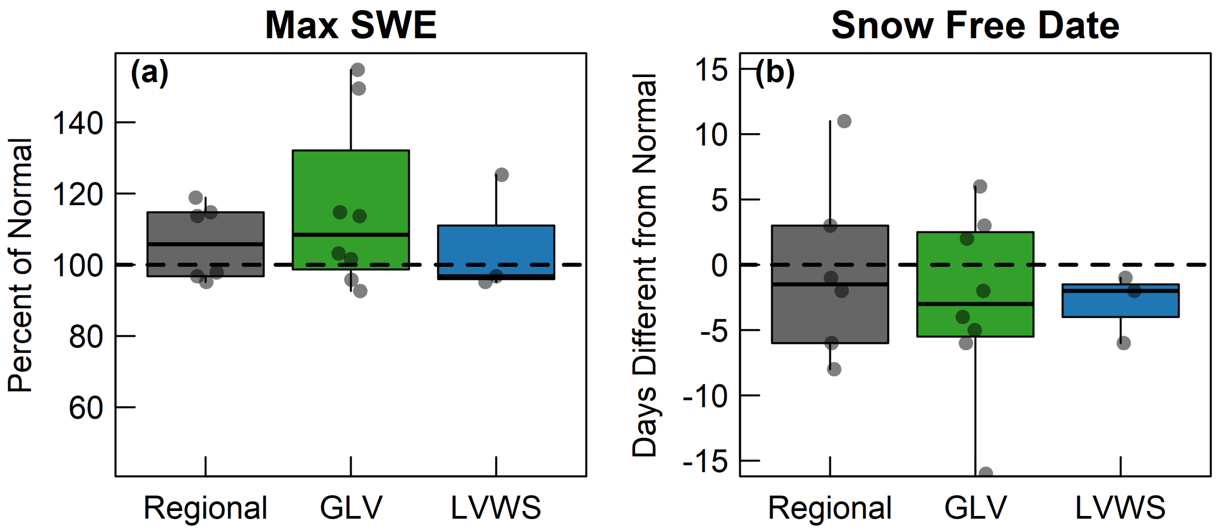

Figure S8. Boxplots displaying maximum snow water equivalent (SWE) as a percent of normal (1980-2010) and the difference in the first snow free date as compared to normal (1980-2010). Data for the GLV and LVWS models were based on a single SNOTEL site for each model with data points representing a different year. Because many of the lakes in the All Lakes model are located close together, often times a single SNOTEL site represents multiple lakes. In general, the period of observation represented in these models had slightly above normal SWE but somewhat early melt-off.

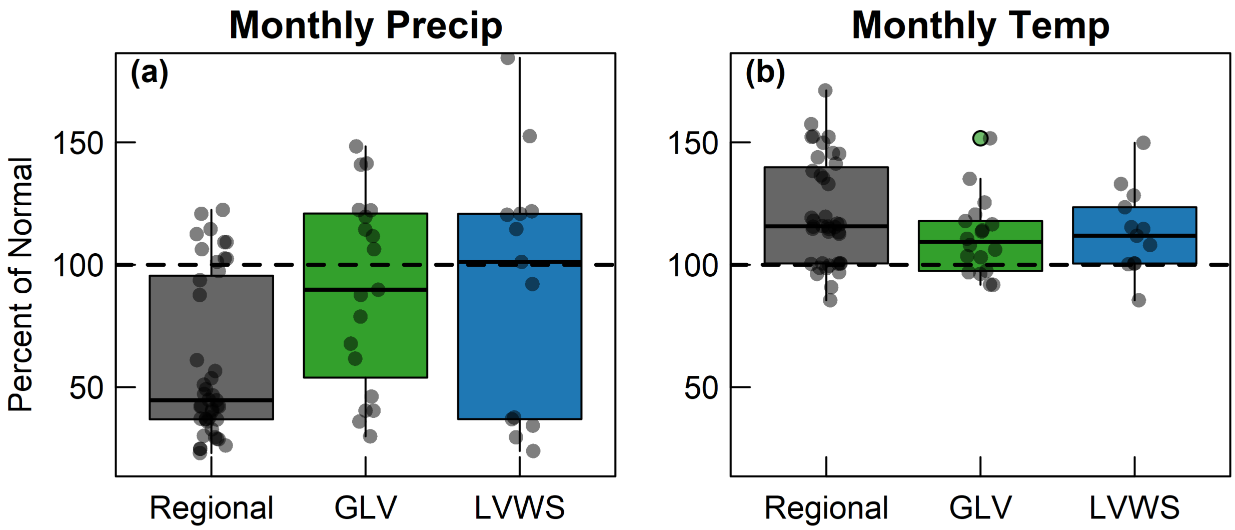

Figure S9. PRISM climate data of monthly precipitation and temperature as a percent of normal (1980-2010). Generally, the period of observation was drier and hotter than normal. Median values for monthly precipitation were 45%, 90%, and 101% of the 30-year average (Regional, GLV, and LVWS, respectively). Median values for monthly temperature were 116%, 109%, and 112% of the 30-year average (Regional, GLV, LWVS, respectively).
