# Supplementary material for: The role of warm, dry summers and variation in snowpack on phytoplankton dynamics in mountain lakes
Source: Ecology. 2020 Sep 16;101(10):e03132. doi: 10.1002/ecy.3132 (PMC7583380; doi:10.1002/ecy.3132)
Supplement: Supplementary file 3 — Appendix S3 [file ECY-101-e03132-s003.pdf]

Supporting Information. Oleksy, I.A., W.S. Beck, R.W. Lammers, C.E. Steger, C. Wilson, K. Christianson, K. Vincent, G. Johnson, P.T.J. Johnson, and J.S. Baron. 2020. The role of warm, dry summers and variation in snowpack on phytoplankton dynamics in mountain lakes. Ecology.

Appendix S3 – Linear mixed models

Linear mixed-effects models (LMMs) were created for each of the three datasets in the *lme4* package (Bates et al. 2015). Fixed effects for each LMM included predictors with the five highest V.I. scores, as determined by the best BRTs. We included "lake identity" (regional model) and "sampling event" (all models) as random effects to account for correlations among observations that were collected in the same lake or on the same date. The *MuMIN* package (Barton 2019) was used to calculate conditional R_2 values (R_{2c}) for each model, which represent only the variability explained by fixed effects, and marginal R_2 values (R_{2m}), which represent the variability explained by fixed and random effects in the model. We used *lmerTest* (Kuznetsova et al. 2017) to calculate p-values for each of the fixed effects.

The regional LMM included the top five variables from the Regional Climate model as fixed effects, along with sampling event and lake identity as random effects. It had a very low R_{2c} of 0.024 and moderate R_{2m} of 0.451 (Table S1). None of the fixed effects were significant predictors in the model (all $p > 0.05$). Sampling event explained more variation in chlorophyll *a* than did lake identity as a random effect. The Long-term LMM including the top five variables from the Long-term BRT model as fixed effects, along with sampling date as a random effect,

had a strong R^2_c of 0.76 and R^2_m of 0.90 (Table S1). Maximum SWE was negatively related with chlorophyll *a* (p<0.001). Additionally, while the effect of NO_3 alone was not statistically significant, the interaction between NO_3*year (p=0.004) and NO_3*Max. SWE had a significant positive effect on chlorophyll *a* (p<0.001). The Intra-seasonal LMM including the top five variables from the Intra-seasonal BRT model as fixed effects, along with sampling date as a random effect, had a moderate R^2_c of 0.52 and strong R^2_m of 0.72. The molar ratio of DIN to TP and water temp. had significant negative relationships with chlorophyll *a* (p=0.001 and p=0.015, Table S1). In both lakes, the highest chlorophyll *a* concentrations were observed late in the open-water season several weeks after ice-off when DIN:TP was lowest and water temperatures in each lake were warmest

Table S1. Results of linear mixed effects models for each of the three datasets, which included the most important boosted regression tree variables as fixed effects. The Regional Climate model included sampling event and lake identity as random effects, and the Long-term and Intra-seasonal models included sampling date as a random effect. R^2_m refers to the marginal R^2 value incorporating variance explained by fixed effects only. R^2_c refers to the conditional R^2 value incorporating variance explained by fixed and random effects. P-values <0.05 are denoted as "*".

Model	Predictor	Est.	SE	df	t-val	p-val	R^2_m	R^2_c
**Regional**							**0.024**	**0.451**
	Intercept	-0.076	1.344	44.520	-0.057	0.955		
	Weekly precip.	0.001	0.014	36.831	0.082	0.935		
	Daily mean temp.	0.0109	0.0549	34.020	0.199	0.844		
	Monthly mean temp.	0.022	0.063	37.219	0.354	0.725		
	Mean temp. % normal	-0.052	0.498	46.457	-0.105	0.917		
	DOY	0.005	0.005	46.422	1.012	0.317		
**Long-Term**							**0.760**	**0.903**
	Intercept	1.398	0.213	46.260	6.553	<0.001*		
	NO3	-7.595	4.967	50.321	-1.529	0.133		
	Max. SWE	-0.430	0.050	42.545	-8.677	<0.001*		
	Water temp.	-0.115	0.099	73.831	-1.154	0.252		
	DIN:TDP	0.000	0.000	70.258	0.354	0.725		
	Daily precip.	0.015	0.038	39.088	0.384	0.703		
	Year	-0.087	0.104	51.340	-0.840	0.405		
	NO3* Year	6.141	2.057	49.500	2.985	0.004*		
	NO3 * Max. SWE	6.580	0.908	52.405	7.244	<0.001*		
**Intra-Seasonal**							**0.517**	**0.718**
	Intercept	-0.186	1.225	56.501	-0.152	0.880		
	DOY	0.011	0.003	43.602	3.240	0.002*		
	Monthly precip.	0.000	0.003	31.098	-0.008	0.993		
	Weekly mean temp.	0.068	0.045	43.629	1.497	0.142		
	Water temp.	-0.104	0.031	66.383	-3.386	0.001*		
	DIN:TP	-0.013	0.005	68.476	-2.502	0.015*		

Literature cited

Bates, D., Maechler, M., Bolker, B., Walker, S. (2015). Fitting linear mixed-effects models using lme4. *Journal of Statistical Software*, *67*(1), 1-48.doi:10.18637/jss.v067.i01.

Barton, K. (2019). MuMIn: Multi-Model Inference. R package version 1.43.6. https://CRAN.R-project.org/package=MuMIn

Kuznetsova, A., Brockhoff, P.B., Christensen, R.H.B. (2017). *lmerTest package: Tests in linear mixed effects models*. *Journal of Statistical Software*, *82*(13), 1-26. https://doi.org/10.18637/jss.v082.i13.
